# Multiple nutrient transporters enable cells to mitigate a rate-affinity tradeoff

**DOI:** 10.1371/journal.pcbi.1010060

**Published:** 2022-04-25

**Authors:** Luis Fernando Montaño-Gutierrez, Kevin Correia, Peter S. Swain

**Affiliations:** School of Biological Sciences, University of Edinburgh, Edinburgh, United Kingdom; North Carolina State University, UNITED STATES

## Abstract

Eukaryotic genomes often encode multiple transporters for the same nutrient. For example, budding yeast has 17 hexose transporters (HXTs), all of which potentially transport glucose. Using mathematical modelling, we show that transporters that use either facilitated diffusion or symport can have a rate-affinity tradeoff, where an increase in the maximal rate of transport decreases the transporter’s apparent affinity. These changes affect the import flux non-monotonically, and for a given concentration of extracellular nutrient there is one transporter, characterised by its affinity, that has a higher import flux than any other. Through encoding multiple transporters, cells can therefore mitigate the tradeoff by expressing those transporters with higher affinities in lower concentrations of nutrients. We verify our predictions using fluorescent tagging of seven HXT genes in budding yeast and follow their expression over time in batch culture. Using the known affinities of the corresponding transporters, we show that their regulation in glucose is broadly consistent with a rate-affinity tradeoff: as glucose falls, the levels of the different transporters peak in an order that mostly follows their affinity for glucose. More generally, evolution is constrained by tradeoffs. Our findings indicate that one such tradeoff often occurs in the cellular transport of nutrients.

## Author summary

From yeast to humans, cells often express multiple different types of transporters for the same nutrient, and it is puzzling why a single high-affinity transporter is not expressed instead. Here we initially use mathematical modelling to demonstrate that transporters facilitating diffusion and those powered by the proton motive force can both exhibit a rate-affinity tradeoff, for quite general conditions. A transporter with a higher affinity necessarily has a lower rate, and vice versa. The tradeoff implies that there is a range of nutrient concentrations for which a transporter, characterised by its affinity, has a higher import flux than any other transporter with a different affinity. To mitigate the tradeoff, genomes may therefore encode multiple different transporters, and cells that express each transporter in the concentrations where it imports best will uptake nutrients at higher rates. Consistently, we show that as cells of budding yeast consume glucose, they express five types of hexose transporters in an order that follows the transporters’ affinities.

## Introduction

To grow and divide, cells must import nutrients, and genomes often encode several types of transporters for the same nutrient. In the budding yeast *Saccharomyces cerevisaie*, for example, multiple transporters may be the norm rather than the exception, particularly for essential nutrients—there are two transporters for sulphate [1], five for phosphate [2], three for ammonium [3], and remarkably up to 18 for glucose [4]. Similarly, the human genome encodes 14 transporters for glucose that, like yeast’s, use facilitated diffusion [5] and six more that are symporters powered by the sodium motive force [6]. We also express at least six different phosphate transporters in the kidney [7].

It is puzzling why multiple transporters have been selected. Why not have one type of transporter with a high affinity that imports as fast as possible? For nutrient sensing in budding yeast, several explanations have been proposed.

One possibility is that by having a low and a high affinity transporter for a nutrient, cells are better able to prepare for that nutrient becoming scarce [8]. As the nutrient’s availability falls, cells use the drop in flux through the low affinity transporter as a warning to trigger expression of the high affinity one. Cells therefore maintain intracellular nutrients long enough to be able to launch a preparatory programme of gene expression before extracellular nutrients are depleted [8].

Another possibility, at least for transporters using facilitated diffusion, is that levels of transporters are regulated to reduce the efflux of valuable nutrients [9]. If the intracellular concentration of a nutrient rises in a medium rich in the nutrient, then a high affinity transporter will be bound by the nutrient both intracellularly and extracellularly and will no longer import. By expressing a transporter with a lower affinity, cells will have a transporter that is not saturated intracellularly, enabling import to continue and intracellular nutrient to accumulate [9]. In yeast, intracellular concentrations of glucose can become high [10], but this argument may not hold for active transporters, which should rarely export their substrate.

Perhaps the most general possibility is that the transporters have a rate-affinity tradeoff [11]. Increasing a transporter’s maximal rate of import of a nutrient may necessarily decreases its affinity for the nutrient. Such a tradeoff would support having more than two transporters and may hold irrespective of the transport mechanism. Cells would encode multiple transporters with different affinities to mitigate the tradeoff. Transporters with lower affinities and higher rates would be expressed when nutrient concentrations are high, and transporters with higher affinities and lower rates would be expressed when levels of nutrients are low.

Here, like others [9], we use mathematical modelling to determine if such rate-affinity tradeoffs are possible in principle, focusing on facilitated diffusion, but considering symporters too. We show that both types of transport do exhibit a tradeoff for quite general conditions and consequently that for multiple transporters, there is a range of concentrations of extracellular substrates where one transporter performs better than any other. Using fluorescent proteins to follow the levels of seven hexose transporters in budding yeast, we demonstrate that the order of their peaks in expression as glucose falls from high to low concentrations is broadly consistent with their measured affinities and a rate-affinity tradeoff.

## Results

### Facilitative transporters can have a rate-affinity tradeoff

**Fig 1:**
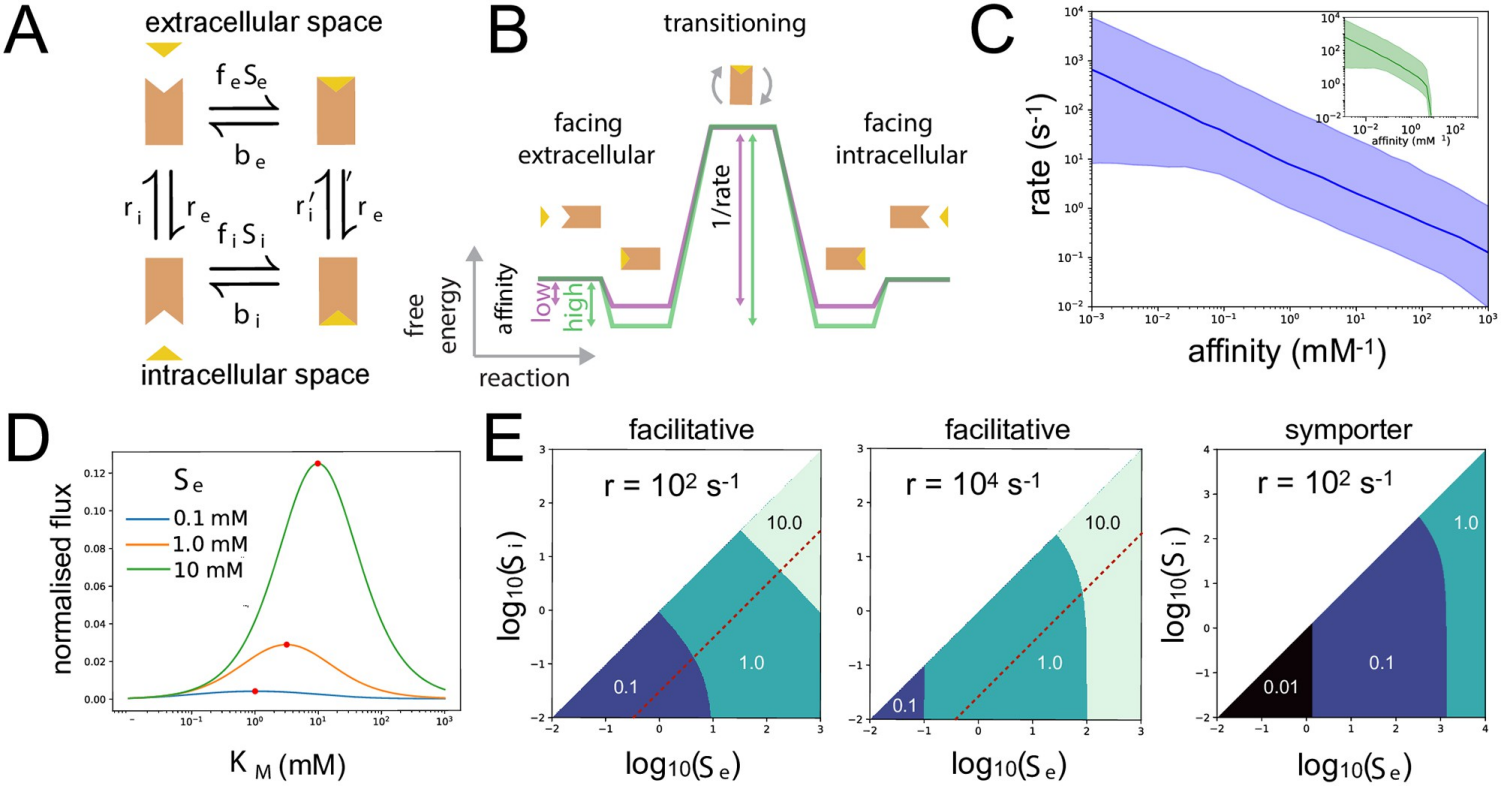
Transport by facilitated diffusion can exhibit a rate-affinity tradeoff. **A.** Transport by facilitated diffusion is driven by thermal fluctuations causing the transporter to re-orient continually to face either the extracellular space or the cytoplasm. We denote extra- and intracellular substrate as *S*_*e*_ and *S*_*i*_ (yellow triangle), the association rate of extracellular substrate by *f*_*e*_ and its dissociation rate by *b*_*e*_, the association rate of intracellular substrate by *f*_*i*_ and its dissociation rate by *b*_*i*_, and the transporter’s rate of transitioning across the membrane by *r*^′^ when bound by substrate and *r* otherwise. **B.** The rate-affinity tradeoff may be understood from a reaction coordinate diagram. High affinity transporters (green) necessarily have a lower rate than low affinity transporters (purple). **C.** Randomly sampling *b*_*e*_, *f*_*e*_, *f*_*i*_, and $r=r_{i}^{\prime }=r_{e}^{\prime }$ (*b*_*i*_ is given by Eq 3) reveals the tradeoff by plotting the median rate, via Eq 5, against the median affinity, via Eq 6. The shading shows the interquartile range. Here *S*_*e*_ has a concentration of 10 mM, and *S*_*i*_ is either 10^−5^ *S*_*e*_ (blue) or 10^−2^ *S*_*e*_ (green inset). A larger *S*_*i*_ worsens the tradeoff. **D.** For a given *S*_*e*_ and *S*_*i*_, there is a transporter—characterised by its apparent *K*_*M*_ and denoted with a red dot—that maximises import. As *S*_*e*_ increases so too does the optimal *K*_*M*_. We change *K*_*M*_ by varying *b*_*e*_ and set *S*_*i*_ = 10^−5^ *S*_*e*_, *r* = *r*^′^ = 10^4^ s^−1^, *f*_*e*_ = 10^6^ mM^−1^ s^−1^ (diffusion-limited [12]), and *f*_*i*_ = 10^−3^ *f*_*e*_. The flux is normalised by *r*. The optimal *K*_*M*_ changes little if *S*_*i*_ is increased. **E.** If cells have multiple transporters that differ only in their *K*_*d*_, then to maximise flux each should be expressed for a characteristic range of the extracellular and intracellular concentrations of substrate. We consider three transporters with a *K*_*d*_ of either 0.01, 0.1, 1, or 10 mM, and *b*_*e*_ is calculated from this *K*_*d*_ value. Shading shows the region where a particular transporter is optimal: each region is labelled by the corresponding *K*_*d*_ with darker colours corresponding to lower values. Concentrations are in mM, and *f*_*e*_ = 10^6^ mM^−1^ s^−1^. For facilitative transporters, *f*_*i*_ = *f*_*e*_/10^3^. The regions are more determined by *S*_*e*_ alone if *r* is larger because the time available for a cytoplasmic substrate to bind the receptor is then reduced. Illustrative lines where *S*_*i*_ is proportional to *S*_*e*_ are shown with red dashes. For the symporter, *f*_*i*_ = *f*_*e*_/10^2^, *m* = *n* = 1, *z*_*S*_ = 0, Δ*ψ* = −100 mV, the extracellular pH is 5, the intracellular pH is 7, and λ = 0.3 (Materials and methods).

Consider a transporter that uses facilitated diffusion (Fig 1A). Embedded in the plasma membrane, its structure randomly fluctuates—facing inwards towards the cytoplasm, outwards towards the extracellular space, and then back again—and so it passively transports substrates from high to low concentrations.

The transporter’s potential for a rate-affinity tradeoff may be understood intuitively using a reaction coordinate diagram (Fig 1B) [13]. Its affinity is determined by the difference in free energy between a substrate in solution and one bound to the transporter—the larger the free energy difference, the higher is the affinity. Its rate of import is mainly determined by the difference in free energy between the substrate-bound form and the transition state as the transporter changes to face the intracellular space—the larger the free energy difference, the lower is the import rate, because the activation barrier is greater. Assuming that the time to cross this barrier is substantially longer than the time for the substrate to unbind from the transporter and enter the cytosol, then an increase in affinity necessarily decreases the rate.

It is straightforward to calculate the transporter’s steady-state flux [14]—a Michaelis-Menten function of the difference between the extra- and intracellular concentrations of substrate. Assuming that the membrane is sufficiently symmetrical that the rates of an unbound transporter’s transitioning across it are similar in both directions, *r*_*i*_ = *r*_*e*_ = *r* (Fig 1A), that the substrate is uncharged so that $r_{i}^{\prime }=r_{e}^{\prime }=r^{\prime }$ too for a bound transporter, and writing *S*_*e*_ for extracellular substrate, *S*_*i*_ for intracellular substrate, Δ*S* = *S*_*e*_ − *S*_*i*_, we find that

$$\begin{array}{c} J=\frac{K_{d}rr^{\prime }\;\Delta S}{2\left(K_{d}+S_{i}\right)[r\left(K_{d}+\frac{r^{\prime }}{f_{e}}\right)+r^{\prime }\left(S_{i}+\frac{r}{f_{i}}\right)]+[K_{d}\left(r+r^{\prime }\right)+2r^{\prime }\left(S_{i}+\frac{r}{f_{i}}\right)]\;\Delta S}. \end{array}$$ (1)

Here *f*_*e*_ and *f*_*i*_ are the rates of association of substrate to the transporter and *b*_*e*_ and *b*_*i*_ are the rates of dissociation. The extracellular substrate’s dissociation constant of binding, *K*_*d*_, is defined as

$$\begin{array}{c} K_{d}=\frac{b_{e}}{f_{e}}. \end{array}$$ (2)

The rate constants are interdependent [14] and obey

$$\begin{array}{c} \frac{b_{e}}{f_{e}}=\frac{b_{i}}{f_{i}} \end{array}$$ (3)

so that transport is able to reach equilibrium when *S*_*e*_ = *S*_*i*_. Without Eq 3, at least one of the kinetic steps in Fig 1A must be thermodynamically driven through, for example, an effectively irreversible reaction like hydrolysing ATP. We interpret Eq 3 to mean that *b*_*i*_ is a dependent parameter, which we eliminate from Eq 1.

Comparing Eq 1 with

$$\begin{array}{c} J=\frac{k_{\text{cat}}\Delta S}{K_{M}+\Delta S} \end{array}$$ (4)

we can characterise transport with an apparent *k*_cat_ and *K*_*M*_, which depend on *S*_*i*_.

To clarify, and following others [15, 16], when we write *K*_*M*_ we mean the apparent *K*_*M*_ defined by Eq 4. By affinity, we mean the apparent affinity—the reciprocal of this *K*_*M*_. The rate at which a transporter works is its flux, *J* in Eq 1. The maximal rate, or maximal flux, is given by *k*_cat_. The dissociation constant of the extracellular substrate binding to the transporter is *K*_*d*_, and *K*_*M*_ and *k*_cat_ are both functions of *K*_*d*_ and the rate constants in Eq 1, as well as *S*_*i*_.

When we vary a rate constant that determines transport, there is a physiologically relevant rate-affinity tradeoff if two conditions are met: first, the partial derivatives of *k*_cat_ and the affinity, 1/*K*_*M*_, with respect to the rate constant should have opposite signs so that 1/*K*_*M*_ decreases when *k*_cat_ increases and vice versa; second, the partial derivative of the import flux, *J*, with respect to the rate constant should be non-monotonic. For example, if this partial derivative of *J* is always positive, then even if there is a tradeoff, and increasing the rate constant decreases the affinity, the resulting change in the flux is more than compensated by the corresponding increase in *k*_cat_. Mutations increasing the rate constant will always be favoured, making the tradeoff unimportant.

Performing this test—calculating the three partial derivatives and inspecting their signs—with respect to each of the rate constants constituting *J* in Eq 1 and using *b*_*e*_ rather than *K*_*d*_ = *b*_*e*_/*f*_*e*_ and $r_{i}^{\prime }=r_{e}^{\prime }=r^{\prime }$, we find that no physiologically relevant tradeoff is possible through changing *f*_*i*_, *r*, and *r*^′^, with the corresponding partial derivatives of *J* always being positive. Physiologically relevant tradeoffs are possible through changing *b*_*e*_ and *f*_*e*_, the parameters determining the extracellular binding of the substrate, but only if some of the rate constants have their values constrained.

Alternatively, if the transporter transitions across the membrane at the same rate regardless of whether any substrate is bound or not so that *r*^′^ = *r*, implicitly assuming that the transporter’s structure does not substantially change when bound by substrate, then a physiologically relevant tradeoff occurs for all non-zero values of the other rate constants when we vary *b*_*e*_ and *f*_*e*_. When *r*^′^ = *r*

$$\begin{array}{c} k_{\text{cat}}=\frac{\frac{1}{2}K_{d}r}{K_{d}+\frac{r}{f_{i}}+S_{i}} \end{array}$$ (5)

and

$$\begin{array}{c} K_{M}=\left(K_{d}+S_{i}\right)(1+\frac{\frac{r}{f_{e}}}{K_{d}+\frac{r}{f_{i}}+S_{i}}). \end{array}$$ (6)

We note that more intracellular substrate undermines import as expected [9], by both decreasing *k*_cat_ (Eq 5) and increasing *K*_*M*_ (Eq 6). Differentiating Eqs 5 and 6, we find a rate-affinity tradeoff for all parameters (Fig 1C), but, as before, this tradeoff is only physiologically relevant if we vary *b*_*e*_ and *f*_*e*_—both of which determine the extracellular *K*_*d*_. We can show that *k*_cat_ decreases with increasing *f*_*e*_ while the affinity increases, and that *k*_cat_ increases with increasing *b*_*e*_ while the affinity decreases. The corresponding partial derivatives of *J* are non-monotonic as required.

Characterising a transporter by its apparent *K*_*M*_, the tradeoff then implies that there is an optimal transporter that maximises flux for given concentrations of extra- and intracellular substrate. Lowering *K*_*M*_ away from the optimal value—raising the affinity, should increase the flux (Eq 4), but lowering *K*_*M*_ also lowers *k*_cat_. Providing we change *K*_*M*_ by varying *b*_*e*_ or *f*_*e*_, the flux therefore decreases at sufficiently small *K*_*M*_. Raising *K*_*M*_ away from the optimal value also eventually decreases flux too because *k*_cat_ saturates. Consequently, there is a *K*_*M*_ that maximises flux for each *S*_*e*_ and *S*_*i*_ (Fig 1D).

Considering, say, three transporters that differ only in their values of *b*_*e*_, or equivalently in *K*_*d*_ because *K*_*d*_ = *b*_*e*_/*f*_*e*_, then each transporter will be optimal—having the greatest import flux out of all three transporters—for a particular range of values of *S*_*e*_ and *S*_*i*_ (Fig 1E). A higher *S*_*e*_ favours transporters with higher *K*_*d*_, and vice versa. From Eqs 5 and 6, a higher *S*_*e*_ therefore favours transporters with a lower affinity and a higher *k*_cat_. In this regime, transporters are more likely to be saturated, working close to their maximal rate of *k*_cat_ with the affinity little affecting flux. A lower *S*_*e*_ favours transporters with a higher affinity and lower *k*_cat_. Transporters are likely to be far from saturated with the affinity strongly determining flux.

The behaviour in Fig 1D & 1E is distinct from the product inhibition suffered by facilitative transporters for large concentrations of intracellular substrate [9]. If *S*_*i*_ is proportional to *S*_*e*_ so that *S*_*i*_/*S*_*e*_ is a constant fraction, then in the limit of sufficiently large *S*_*i*_, and so too of an even larger *S*_*e*_, Eq 1 becomes

$$\begin{array}{c} J≃\frac{K_{d}r}{2S_{i}}(1-\frac{S_{i}}{S_{e}}). \end{array}$$ (7)

The flux tends to zero as *S*_*i*_ increases further even though *S*_*e*_ > *S*_*i*_ is also increasing. With a large enough *S*_*i*_, any substrate molecule that dissociates from the transporter to enter the cytoplasm is almost always replaced by another cytoplasmic substrate before the transporter reorients to face the extracellular space. This product inhibition will occur irrespective of whether or not there is a rate-affinity tradeoff. Setting *S*_*i*_ to be proportional to *S*_*e*_ corresponds to moving along a line in Fig 1E that is parallel to the diagonal and intercepts the *x*-axis at negative values. Although the ranges of *S*_*e*_ for which each transporter is optimal alter along such a line compared to when *S*_*i*_ is negligible (*x*-axis), the order in which the different transporters become optimal as *S*_*e*_ changes remains the same.

To summarise, we find that transporters using facilitated diffusion always exhibit a rate-affinity tradeoff if their rate of transitioning back and forth across the membrane is symmetric and unchanged when bound by substrate. This tradeoff affects flux only if the maximal rate and affinity are changed by varying the rate constants that determine the extracellular *K*_*d*_, *b*_*e*_ and *f*_*e*_. Flux increases monotonically with the other rate constants, *f*_*i*_ and *r*, which we might assume have values close to the maxima that selection allows.

### Symporters can also have a rate-affinity tradeoff

Many transporters work not by facilitated diffusion but are powered by the proton motive force across the plasma membrane. For example, in budding yeast, most phosphate [2] and amino acid transporters [2, 17] are proton symporters. Despite being driven by the proton motive force, we show that symporters too may have a rate-affinity tradeoff.

Symporters can be modelled as facilitative transporters [14] (Materials and methods). By replacing the binding of the substrate in Fig 1A with the binding of *m* protons and *n* substrates and assuming that the intermediate states with only some of the protons and substrates bound are short-lived, then the steady-state flux of imported substrate becomes similar to Eq 1. The rates *r*^′^ and *b* in Fig 1A now describe the transport rates of protons and substrates through the symporter; the rate *r* determines a refractory period, where the symporter is temporarily unable to transport.

As before, we define an apparent *k*_cat_ and *K*_*M*_ to describe the transport. Writing *S* for a substrate molecule, we can rearrange the expression for the antiporter’s steady-state flux to have the form

$$\begin{array}{c} J=\frac{k_{\text{cat}}\Delta S_{\text{sym}}}{K_{M}+\Delta S_{\text{sym}}} \end{array}$$ (8)

where Δ*S*_sym_ is

$$\begin{array}{c} \Delta S_{\text{sym}}=e^{-u}{\left[H^{+}\right]}_{e}^{m}S_{e}^{n}-{\left[H^{+}\right]}_{i}^{m}S_{i}^{n} \end{array}$$ (9)

and

$$\begin{array}{c} u=\frac{F}{RT}\left(m+nz_{S}\right)\Delta \psi . \end{array}$$ (10)

Here Δ*ψ* denotes the plasma membrane potential, *R* is the ideal gas constant, *T* is temperature, *F* is Faraday’s constant, and a substrate molecule has charge *z*_*S*_.

The apparent *k*_cat_ and *K*_*M*_ are

$$\begin{array}{c} k_{\text{cat}}=\frac{r}{1+e^{(1-\lambda )u}+(1+e^{u})C} \end{array}$$ (11)

and

$$\begin{array}{c} K_{M}=(2K_{d}+(1+e^{u}){\left[H^{+}\right]}_{i}^{m}S_{i}^{n})\times \frac{e^{-\lambda u}+e^{-u}(\frac{r}{b_{e}}+e^{u}\;C)}{1+e^{(1-\lambda )u}+(1+e^{u})C} \end{array}$$ (12)

writing

$$\begin{array}{c} C=\frac{\frac{r}{f_{i}}+{\left[H^{+}\right]}_{i}^{m}S_{i}^{n}}{K_{d}} \end{array}$$ (13)

and for a constant λ with 0 ≤ λ ≤ 1 (Materials and methods). By calculating the derivatives of Eqs 11 and 12 and of the flux, Eq 8, we can check for physiologically relevant tradeoffs when any one of the parameters describing transport is varied.

Although the expressions for the derivatives are complicated preventing a complete analysis, we find that it is possible to have physiologically relevant rate-affinity tradeoffs despite transport being powered by the proton motive force. Although the symporter’s flux always increases with increasing *r* or *f*_*i*_, there is such a tradeoff when *f*_*e*_ changes and also when *b*_*e*_ changes if *f*_*e*_ ≥ *f*_*i*_. This inequality favours import and so is likely to be obeyed by most nutrient symporters. Even if substrate binding disfavours import, there is still a tradeoff when *b*_*e*_ changes if *u* is sufficiently large and negative such that *e*^*u*^ ≪ 1. Because Δ*ψ* is typically negative [14], this condition is most likely met for positively charged substrates or requires multiple protons to be co-transported with each negative substrate so that *m* > −*nz*_*S*_. In all these cases, the derivative of the flux may be non-monotonic, implying that such a symporter, like a facilitative transporter, has particular extracellular conditions where its flux is maximal (Fig 1E).

### Expression of hexose transporters is consistent with a rate-affinity tradeoff

Given these results, we considered glucose transport in budding yeast to determine if such a tradeoff is consistent with the expression of yeast’s genes for hexose transporters. Although there are 17 HXT genes, seven, encoded by HXT1–7, are thought to be most important for growth on glucose [18]. Each transporter uses facilitated diffusion [19] and has a different apparent affinity for glucose [16, 20], with their levels peaking at different glucose concentrations [21–26]. Focusing on HXT1–7, we tagged these genes with Green Fluorescent Protein (GFP) and followed their levels in cells over time using a microplate reader (Materials and methods).

**Fig 2:**
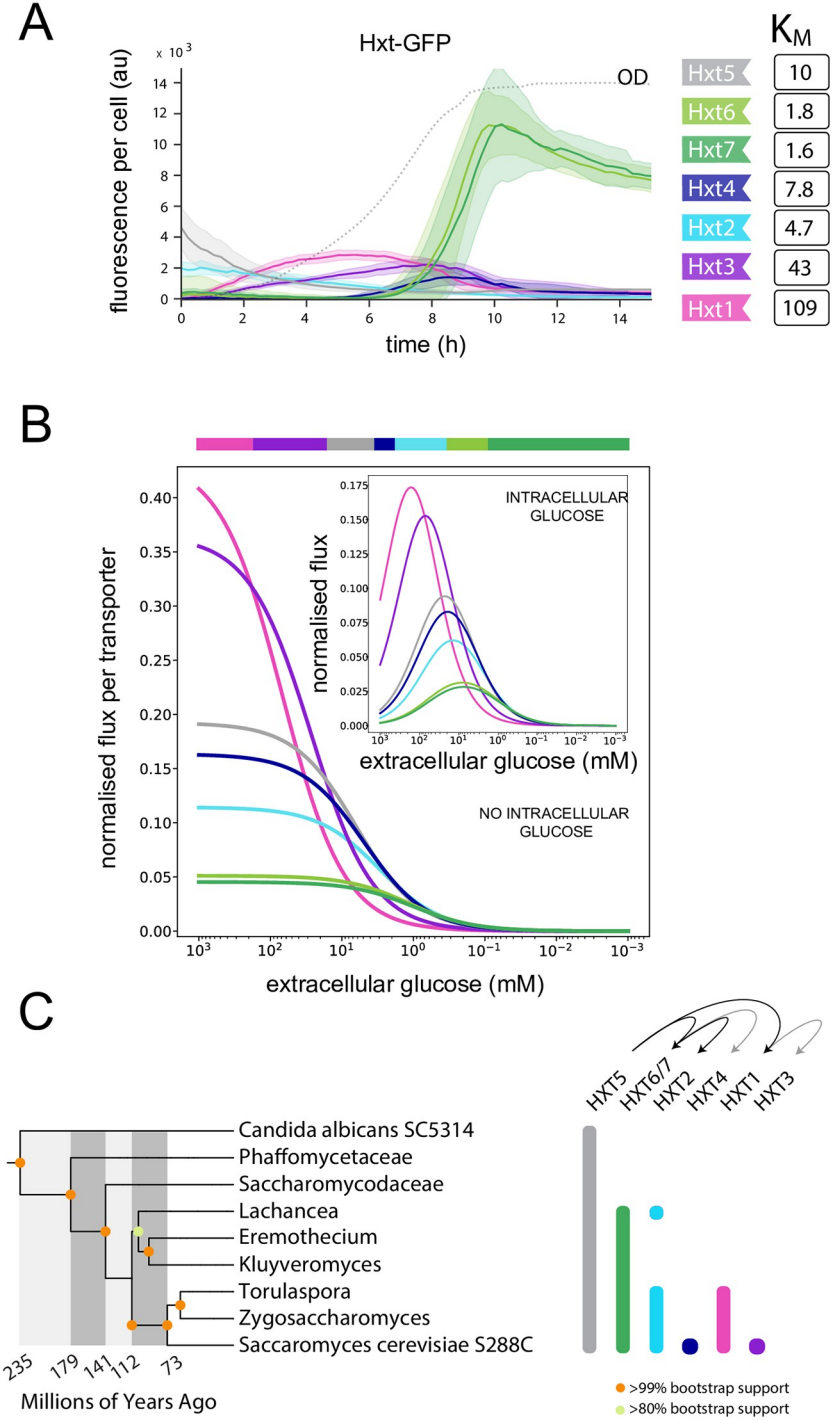
Regulation of yeast’s hexose transporters is broadly consistent with transport having a rate-affinity tradeoff. **A.** As glucose falls, the different HXTs are expressed in an order approximately determined by their *K*_*M*_. We follow transporters tagged with GFP in batch culture with initially 2% (110 mM) glucose and show the mean fluorescence per cell. The concentration of glucose falls as the culture’s optical density (OD) increases (dotted line) and is near zero when the OD plateaus. The shaded regions indicate 95% confidence intervals found using bootstrapping over five replicate experiments. **B.** Using the apparent *K*_*M*_ and assuming that cells optimise import, we can predict the order of expression of the HXTs in falling glucose. The main panel shows predicted import fluxes when intracellular glucose is zero, with the upper bar indicating which transporter has the highest flux. For the inset, the concentration of intracellular glucose is proportional to extracellular glucose (set at 20%), but the order of the optimal transporters in falling glucose is unchanged (compare the maximal flux in the inset to the upper bar). Fluxes are normalised by *r* = 10^4^ s; *f*_*e*_ is at the diffusion limit of 10^6^ mM^−1^ s^−1^ [12]; *f*_*r*_ = *f*_*e*_/10^3^. **C.** A phylogenetic analysis suggests that the newly duplicated HXT genes evolved affinities for novel ranges of glucose concentrations. We show the phylogenetic tree based on 11 orthologous proteins for nine species of yeast. For each HXT gene, the vertical bars show those species whose genome encodes that gene. HXT5 is likely the ancestor of all the HXTs because it is the only one present in all nine species. The arrows show the likely origins of duplications. Based on the apparent affinities in *S. cerevisiae*, HXT5, with medium affinity, gave rise to the high affinity HXT6/7 and to the low affinity HXT1. High affinity HXT6/7 gave rise to the medium affinity HXT2 and HXT4. Very low affinity HXT1 gave rise to the low affinity HXT3.

For cells in 2% glucose, we observed that the transporters are mostly expressed sequentially in time following their apparent affinity (Fig 2A). The low affinity transporters—Hxt1 and then Hxt3—peak first when the concentration of glucose is highest, followed by the medium affinity transporter Hxt4, and then the high affinity transporters Hxt6 and Hxt7, which peak as glucose is exhausted. There are two exceptions: the medium affinity transporters Hxt2 and Hxt5, but both are known to be atypical Hxts. Hxt2 is unusual because only its mRNA is enriched in the buds that form when glucose is added to starving cells [27]. HXT5 is regulated differently from the others [28, 29], and levels of the corresponding transporter are known to decrease monotonically in glucose [30]. With our pre-growth on pyruvate, its behaviour is as expected, with levels that are initially high and fall over time.

Transport of glucose by the Hxts is likely symmetric [20], as we assume in Eqs 5 and 6, and the apparent *K*_*M*_s have been determined [16, 20]. We interpret these estimates to be at negligible intracellular glucose, which was not measured, because the strains used were generated from the HXT-null mutant [31] and expressed only one of HXT1–7. These strains likely have substantially reduced import because the transporters are normally co-expressed in the wild-type strain (Fig 2A). This assumption, though, is for convenience, and our analysis would be similar if intracellular glucose had been measured. The results hinge not on the intracellular glucose but on the different characteristics of the transporters.

Assuming then negligible intracellular glucose for the measured *K*_*M*_s, we can use their values to find a relationship between the *K*_*d*_ of the corresponding transporters and the other rate constants, via Eq 6 with *S*_*i*_ = 0. Fixing *r* and *f*_*i*_—flux increases monotonically with both and so we assume they have the maximal values allowed by selection and are the same for each Hxt—and further assuming, for simplicity, that *f*_*e*_ is diffusion-limited, we can determine a *K*_*d*_ for each transporter and so an apparent *K*_*M*_, *k*_cat_, and flux for non-zero intracellular glucose (Fig 2B). As expected, transporters with a higher apparent *K*_*M*_ have a higher *K*_*d*_ and a higher *k*_cat_: Hxt1 with the smallest affinity has the highest *k*_cat_, and Hxt7 has the lowest.

From these fluxes, we are able to predict the order in which the transporters should be expressed as extracellular glucose falls if the cell always favours the transporter that imports best (Fig 2B bar). For each extracellular concentration of glucose, one Hxt is predicted to maximise the flux of imported glucose, and correspondingly each Hxt has a range of glucose concentrations where it should be optimal. Although the concentrations defining this range depend on our choice of parameters, the order of expression as a function of the extracellular glucose concentration does not. This order is broadly consistent with the observed peak of each Hxt’s expression over time (Fig 2) and so with the Hxts exhibiting a rate-affinity tradeoff.

If intracellular glucose is proportional to extracellular glucose, we see that the flux peaks and then decreases at sufficiently high extracellular glucose because of product inhibition (Fig 2B inset) [9]. As expected (Fig 1E), this inhibition by intracellular glucose does not change the predicted order of expression from when intracellular glucose is negligible (compare the maximal flux of the inset with the upper bar in Fig 2B).

The HXT genes evolved by gene duplication [32], and from a phylogenetic comparison (Fig 2C), we find that the HXT genes arose from a gene related to HXT5 through multiple duplications. If the Hxts do have a rate-affinity tradeoff, then we might expect that the original transporter’s *f*_*e*_ and *b*_*e*_ were selected to maximise import for a particular range of glucose concentrations. For a newly duplicated HXT gene, the *f*_*e*_ and *b*_*e*_ of its transporter presumably evolved to shift its optimal range into a new regime of glucose concentrations. Based on the measured affinities for budding yeast, there is evidence of these transitions: four out of the five duplication events (arrows in Fig 2C) likely led to a transporter with a broadly different affinity.

## Discussion

Using mathematical modelling, we have shown that both passive and active transport can have a rate-affinity tradeoff and that this tradeoff favours the evolution of multiple transporters if selection is for the rapid import of the transporters’ substrate. Once the gene of such a transporter is duplicated, its *K*_*d*_ for substrate may evolve, changing the transporter’s rate and affinity in opposite ways. If its affinity increases, the new transporter can generate a greater import flux than the original transporter at sufficiently low concentrations of substrate because the increase in affinity dominates the decrease in rate; if its affinity decreases, the transporter can generate a greater import flux at sufficiently high substrate concentrations because the increase in the rate dominates the decrease in affinity (Fig 2B). To increase their import of the substrate, cells must further evolve regulation to ensure that as extracellular substrate changes flux is mostly generated by the transporter that is best for the current concentration. For the HXT genes in budding yeast, the result appears analogous to changing gears in a bicycle, with cells seemingly matching the predominant type of transporter to the concentration of glucose available: the order of maximal expression of at least five HXT genes follows the order of their affinities.

A rate-affinity tradeoff does not preclude other explanations for the existence of multiple transporters. We expect that cells use changes in import flux as a warning signal [8] and favour lower affinity facilitative transporters if intracellular concentrations of substrates become too high [9]. Both of these explanations though emphasise sensing of intracellular rather than extracellular nutrients—feedback rather than feedforward control—with its attendant delays. At least for glucose, budding yeast do sense extracellular concentrations [4] and competition for glucose is thought to be fierce [33], likely favouring a rapid response.

Further, cellular decision-making is complex [34], and if optimal, as we have assumed, likely at best Pareto optimal, with multiple competing goals [35]. We know that cells express proteins not only for current conditions but in anticipation of future events [36] and that cells too hedge their bets, suffering an immediate loss in fitness for a potential gain in the future [37]. Genes may also be pleiotropic with additional regulatory constraints. For example, some of yeast’s hexose transporters bind galactose as well as glucose allowing cells to respond to the ratio of the two sugars [38].

Evolution is limited by constraints, and identifying these constraints illuminates our understanding of biology. Here we have argued that one such constraint is likely to be a rate-affinity tradeoff in the cellular transport of nutrients.

## Materials and methods

### Differentiation

All derivatives were calculated using Mathematica (Wolfram Research). Notebooks are available at https://swainlab.bio.ed.ac.uk and as S1 Notebook (facilitative transport) and S2 Notebook (symport).

### Sampling

To generate Fig 1C, we sample uniformly in log space assuming 0.1 < *b*_*e*_ < 10^9^ s^−1^, 10^3^ < *f*_*e*_, *f*_*i*_ < 10^9^ M^−1^ s^−1^, and 0.1 < *r* < 10^7^ s^−1^. Eq 3 gives *b*_*i*_.

### Interdependencies between the rate constants of a symporter

Cells maintain an electrical potential difference across their plasma membranes [14], and because protons and potentially the substrate are charged, the rate at which protons and substrates cross the membrane through the symporter cannot be the same in both directions. Let $T_{e}^{\prime }$ denote a symporter with its *n* protons and *m* substrates at the extracellular space, and $T_{i}^{\prime }$ denote the same symporter with its protons and substrates at the intracellular space. We can represent transport across the membrane by the reaction

$$\begin{array}{c} T_{e}^{\prime }\overset{r_{e}^{\prime }}{\underset{r_{i}^{\prime }}{⇌}}T_{i}^{\prime }, \end{array}$$ (14)

which has a Δ*G* of

$$\begin{array}{c} \Delta G=-RT\;\text{log}\;(\frac{T_{e}^{\prime }}{T_{i}^{\prime }})+F\left(m+nz_{S}\right)\Delta \psi . \end{array}$$ (15)

The substrate has charge *z*_*S*_ so that *m* + *nz*_*S*_ is the total charge transported.

Once we include the effects of both Δ*ψ* and any differences in concentration, this transport step should be able to reach equilibrium. Then the reaction’s Δ*G* is zero implying

$$\begin{array}{c} \frac{T_{e}^{\prime }}{T_{i}^{\prime }}=e^{u} \end{array}$$ (16)

where *u* is defined in Eq 10.

The reaction should also obey detailed balance

$$\begin{array}{c} \frac{T_{e}^{\prime }}{T_{i}^{\prime }}=\frac{r_{i}^{\prime }}{r_{e}^{\prime }}. \end{array}$$ (17)

Together Eqs 16 and 17 imply that

$$\begin{array}{c} \frac{r_{i}^{\prime }}{r_{e}^{\prime }}=e^{u}, \end{array}$$ (18)

so that the difference in rates is determined by the charges of the transported molecules and Δ*ψ* (Eq 10).

To interpret Eq 18, it is helpful to re-parameterise the rates in Eq 18 in terms of *r*, which determines the symporter’s refractory time (Fig 1A). For some constant λ, where 0 ≤ λ ≤ 1, we can write

$$\begin{array}{c} \begin{array}{ccc} r_{i}^{\prime }=re^{\lambda u} & ; & r_{e}^{\prime }=re^{-(1-\lambda )u}, \end{array} \end{array}$$ (19)

which ensures Eq 18 holds. The plasma membrane potential is typically negative [14], and a negative Δ*ψ* should favour the import of positive charge. If the total charge, *m* + *nz*_*S*_, bound to the symporter is positive, then *u* < 0, and Eq 19 implies that $r_{i}^{\prime }<r$ and $r_{e}^{\prime }>r$, as expected.

There are further interdependencies between the rates. Considering now the transport reaction as a whole

$$\begin{array}{c} mH_{e}^{+}+nS_{e}⇌mH_{i}^{+}+nS_{i}, \end{array}$$ (20)

its Δ*G* is

$$\begin{array}{c} \Delta G=-RT\;\text{log}\;(\frac{{\left[H^{+}\right]}_{e}^{m}{\left[S\right]}_{e}^{n}}{{\left[H^{+}\right]}_{i}^{m}{\left[S\right]}_{i}^{n}})+F\left(m+nz_{S}\right)\Delta \psi . \end{array}$$ (21)

Again, this reaction should have the potential to equilibrate. At equilibrium, Δ*G* is zero, implying

$$\begin{array}{c} \frac{{\left[H^{+}\right]}_{e}^{m}{\left[S\right]}_{e}^{n}}{{\left[H^{+}\right]}_{i}^{m}{\left[S\right]}_{i}^{n}}=e^{u} \end{array}$$ (22)

and detailed balance holds so that

$$\begin{array}{c} f_{e}{\left[H^{+}\right]}_{e}^{m}{\left[S\right]}_{e}^{n}r_{e}^{\prime }b_{i}r_{i}=b_{e}r_{e}f_{i}{\left[H^{+}\right]}_{i}^{m}{\left[S\right]}_{i}^{n}r_{i}^{\prime }. \end{array}$$ (23)

Using Eq 22, Eq 23 becomes

$$\begin{array}{c} \frac{b_{e}r_{e}f_{i}r_{i}^{\prime }}{f_{e}r_{e}^{\prime }b_{i}r_{i}}=e^{u} \end{array}$$ (24)

or

$$\begin{array}{c} \frac{b_{e}r_{e}f_{i}}{f_{e}b_{i}r_{i}}=1 \end{array}$$ (25)

because of Eq 18. Setting *r*_*i*_ = *r*_*e*_ = *r*, Eq 25 recovers Eq 3.

With these constraints—Eqs 18 and 25, the symporter’s import flux of substrate *S* can be determined at steady state [14].

### Measured affinities of the Hxts

We use average values for the reported values of the *K*_*M*_s (Table 1).

**Table 1:** Reported values for the apparent *K*_*M*_, the inverse of the affinity, in mM for all Hxts known to be used for growth in glucose. Hxt5, which has distinct regulation [28, 29], has a *K*_*M*_ of 10 mM [28].

Transporter	Methodology				Average
	countertransport [20]	initial uptake [20]	5 mM [16]	100 mM [16]	
Hxt1	107	129	90	110	109
Hxt2	2.9	4.6	1.5	10	4.75
Hxt3	28.6	34.2	55	55	43.2
Hxt4	6.2	6.2	9.3	9.4	7.8
Hxt6	0.9	1.4	2.5	2.5	1.8
Hxt7	1.3	1.9	1.1	2.1	1.6

### Measuring HXT-GFP in budding yeast

#### Strains

To generate HXT-GFP strains, we made individual tags of HXT genes via PCR-based integration of constructs [39]. We chose yEGFP as the fluorophore following previous work [24]. For C-terminal tagging, we obtained plasmids pKT128 [40] from Addgene. We amplified the fluorescent marker cassettes by PCR, transformed the PCR product into yeast, and tested positive colonies by PCR and sequencing.

All strains (Table 2) are derived from strain BY4741 (a derivative of S288C).

**Table 2:** HXT-GFP strains.

Strain ID	In-text description	Genotype
SL229	BY4741	MATa, his3Δ1, leu2Δ0, ura3Δ0, met15Δ0
SL498	HXT1-GFP	SL229 HXT1-yEGFP::HIS
SL480	HXT2-GFP	SL229 HXT2-yEGFP::HIS
SL485	HXT3-GFP	SL229 HXT3-yEGFP::HIS
SL409	HXT4-GFP	SL229 HXT4-yEGFP::HIS
SL487	HXT5-GFP	SL229 HXT5-yEGFP::HIS
SL488	HXT6-GFP	SL229 HXT6-yEGFP::HIS
SL566	HXT7-GFP	SL229 HXT7-yEGFP::HIS

#### Media

We used SC medium (0.2% yeast nitrogen base with 0.5% ammonium sulphate) supplemented with 2% pyruvate for pre-culture. We used low fluorescence SC medium, which is the same as SC medium except riboflavin and folic acid have been removed from the yeast nitrogen base, supplemented with 2% glucose for growth in plate readers.

#### Preparing the cultures

We used 2% pyruvate for pre-culture to avoid any glucose-dependent effects because cells are then respiring and performing gluconeogenesis. We incubated such pyruvate cultures for 48 hours, then diluted and grew cells in fresh medium for another 24 hours, and then, again, diluting and growing in fresh medium for 4 hours to reactivate growth.

#### Measuring OD and fluorescence

We measured optical density and fluorescence in 96 well microplates (Thermo-Fisher) with 200 *μ*l of cell culture using a Tecan M200 plate reader.

#### Analysis and correction of data

We corrected the OD for its non-linear dependence on the number of cells [41] and fluorescence for autofluorescence [42]. We report the mean fluorescence per cell—the corrected fluorescence divided by the corrected OD. All analysis was performed using the omniplate Python module (available from https://swainlab.bio.ed.ac.uk/software/omniplate and https://pypi.org).

### Phylogenetic analysis

We created the species tree (Fig 2C) using a concatenation analysis for *Candida albicans* SC5314, *Cyberlindnera jadinii* NRRL Y-1542 (*Phaffomycetaceae*), *Hanseniaspora valbyensis* NRRL Y-1626 (*Saccharomycodaceae*), *Lachancea thermotolerans* CBS 6340, *Eremothecium gossypii* ATCC 10895, *Kluyveromyces lactis* CBS 2359, *Torulaspora delbrueckii* CBS 1146, *Zygosaccharomyces rouxii* CBS 732, and *Saccharomyces cerevisiae* S288C.

The concatenated sequence was generated using the following proteins, given with the AYbRAH ortholog group in parentheses [43]: Acc1 (FOG02004), Gcn20 (FOG02142), Nup192 (FOG03980), Spb1 (FOG06740), Nup84 (FOG07647), Sec21 (FOG08792), Pom152 (FOG10187), Kap104 (FOG13237), Rpn6 (FOG13362), Rpn1 (FOG13820), and Vps17 (FOG15237). We chose these proteins as they have a strong phylogenetic signal, aligned the sequences with MAFFT—using the default parameters and 10 maximum iterations [44], and reconstructed the phylogenetic tree with IQTree—using the default parameters and 1,000 bootstrap replicates [45]. We estimated divergence times using treePL [46], calibrated with 235 million years of divergence between *Candida albicans* and *Saccharomyces cerevisiae* [47].

We used AYbRAH [43] and the Yeast Gene Order Browser [48] to determine the presence or absence of the hexose transporters orthologs in the taxonomic lineages. Phylogenetic trees were created with Evolview version 2 [49].

## Supporting information

S1 Notebook Mathematica notebook for model of facilitative transport. (NB)

S2 Notebook Mathematica notebook for model of symport. (NB)
